# Case Report: a family report of hereditary pancreatitis caused by SPINK1 and PRSS1 gene mutations

**DOI:** 10.3389/fgene.2025.1610108

**Published:** 2025-09-23

**Authors:** Jing Xu, Ying Hu, Banghui Xiao, Juan He, Miao Zhang, Rui Wang, Nianchun Peng

**Affiliations:** Department of Endocrinology and Metabolism, The Affiliated Hospital of Guizhou Medical University, Guiyang, China

**Keywords:** hereditary pancreatitis, diabetes mellitus, SPINK1 gene, PRSS1 gene, pancreatitis

## Abstract

Hereditary pancreatitis (HP) is a rare genetic disorder of the pancreas. We report a case of a 20-year-old woman presenting with classic features of lean diabetes mellitus, chronic diarrhea, and diabetic ketoacidosis but notably without abdominal pain. Imaging revealed pancreatic calcification. Genetic testing identified pathogenic compound mutations in SPINK1 (c.194+2T>C) and PRSS1 (c.623G>C), confirming a diagnosis of HP. Family screening showed her mother carried a homozygous *SPINK1* mutation, while her siblings variably carried heterozygous *SPINK1* and/or *PRSS1* mutations. All family members with both variants, except the third sister, had pancreatic calcifications. Diabetes was observed in the proband, her mother, and her first and second sisters. This case highlights that in patients under 25 years of age presenting with lean body habitus, diarrhea or steatorrhea, poor islet function, and a family history of diabetes—despite lacking overt abdominal pain—HP should be considered as a differential diagnosis.

## 1 Introduction

Hereditary pancreatitis (HP) was first described in 1952 (Comfort and Steinberg, 1952). It is a rare autosomal dominant genetic disorder associated with mutations in several genes, most notably PRSS1, SPINK1, CFTR, and CTRC (Singh et al., 2019; Panchoo et al., 2022). Although its clinical presentation resembles that of other forms of pancreatitis, HP is distinguished by early onset, frequent abdominal pain, familial inheritance, and an increased risk of diabetes mellitus and pancreatic cancer (Panchoo et al., 2022). Diagnostic criteria include typical pancreatic symptoms, a positive family history, and radiological evidence such as calcifications, ductal dilation, or atrophy observed via computed tomography (CT) or magnetic resonance imaging (MRI) (Singh et al., 2019). We report a case of HP characterized by combined SPINK1 (c.194+2T>C) and PRSS1 (c.623G>C) mutations. This case presented with weight loss and steatorrhea but notably lacked abdominal pain, although a family history of diabetes was present.

## 2 Case presentation

A 20-year-old female patient (the proband, IIa) was admitted to our hospital with complaints of chronic diarrhea and significant weight loss. She reported passing loose, greasy stools 3–5 times daily for 1 year, along with a progressive weight loss of 7 kg. Notably, she denied abdominal pain, mucus, pus, tenesmus, polydipsia, or polyuria. Five days before admission, she visited a local clinic due to worsening diarrhea (5–10 times/day) and was found to have markedly elevated fasting blood glucose levels (>20 mmol/L), prompting referral to our hospital.

**FIGURE 1:**
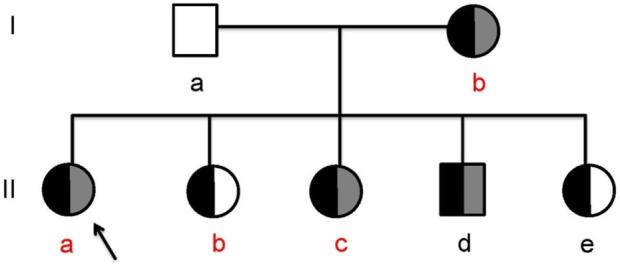
The hereditary pancreatitis family (Ia, the proband’s father; Ib, the proband’s mother; IIa, the proband; IIb, the proband’s first sister; IIc, the proband’s second sister; IId, the proband’s brother; IIe, the proband’s third sister; the black legend, SPINK1 (c.194+2T>C); the gray legend, PRSS1 (c.623G>C); red letter, clinically diagnosed diabetes).

There was no consanguinity between her parents. Her grandparents had died of unknown causes. Her mother (Ib) and eldest sister (IIb) were diagnosed with diabetes mellitus at the ages of 40 and 17 years, respectively. Her second sister (IIc) was diagnosed with diabetes and diabetic ketoacidosis (DKA) at the age of 13. Her third sister (IIe) showed pancreatic atrophy and calcification on imaging but had normal blood glucose levels. Her brother (IId) was asymptomatic and had no abnormal findings (Figure 1; Table 1).

**TABLE 1:** Clinical characteristics and laboratory parameters of this family.

Laboratory parameter	Ia	Ib	IIa (proband)	IIb	IIc	IId	IIe	Reference range	Unit
Sex	M	F	F	F	F	F	M		
Age	46	40	20	17	13	10	8		
Diabetes	N	Y	Y	Y	Y	N	N		
Age at diagnosis	-	40	20	17	10	-	-		
FBG	4.21	8.59	36.56	34.26	34.64	5.41	6.00	3.90–6.10	mmol/L
PBG	5.36	25.69	-	-	-	5.34	5.92		
HbA1c	-	13.5	>15.0	>15.0	19.2	-	-	4.3–5.7	%
C-peptide 0 h	-	72.10	65.51	121.65	0	-	-	279–1,280	pmol/L
C-peptide 2 h	-	319.80	45.02	163.17	53.05	-	-	-	-
Insulin 0 h	-	-	-	-	-	3.47	1.58	3.00–25.00	pmol/L
Insulin 2 h	-	-	-	-	-	12.26	6.27	-	pmol/L
CA199	45.30		37.65					0.00–27.00	U/mL
Serum amylase	-	40.00	43.00	54.2	72.62	-	-	35.00–135.00	U/L
Pancreatic CT	Normal	Pancreatic atrophy and calcification	Pancreatic atrophy and calcification	Normal	Pancreatic atrophy and calcification	Normal	Pancreatic atrophy and calcification		
SPINK1 mutation	-	Homozygous	Heterozygous	Heterozygous	Heterozygous	Heterozygous	Heterozygous		
PRSS1 mutation	-	Heterozygous	Heterozygous	-	Heterozygous	Heterozygous	-	-	
Chronic complications of diabetes	-	Neuropathy	Neuropathy Nephropathy	Unknown	Unknown	-	-		
Treatment	-	Human insulin and glargine	Aspart and glargine	Aspart and glargine	Aspart and glargine	-	-		
5-year follow-up outcome	Normal	Under control	Died	Under control	Died	Normal	Normal		
5-year FBG						5.07		3.90–6.10	mmol/L
5-year PBG						5.26		<7.8	mmol/L
5-year C-peptide 0 h	-	-	-	-	-	656.8	-	279–1,280	pmol/L
5-year C-peptide 2 h	-	-	-	-	-	3337.0	-	-	-
5-year insulin 0 h	-	-	-	-	-	8.79	-	3.00–25.00	pmol/L
5-year insulin 2 h	-	-	-	-	-	109.3	-	-	pmol/L

Ia, the proband’s father; Ib, the proband’s mother; IIa, the proband; IIb, the proband’s first sister; IIc, the proband’s second sister; IId, the proband’s younger brother; IIe, the proband’s third sister; M, male; F, female; N, no; Y, yes; FBG, fasting blood glucose; PBG, postprandial blood glucose; HbA1c, glycated hemoglobin A1c; DKA, diabetic ketoacidosis.

On physical examination, the proband’s height was 153 cm, weight 37 kg, and body mass index (BMI) 15.8 kg/m^2^. Abdominal examination was unremarkable, with no tenderness, rebound pain, or muscular guarding. Diabetic monofilament testing on both feet was positive, though sensory modalities, including pain, temperature, and vibration were intact in the extremities.

**FIGURE 2:**
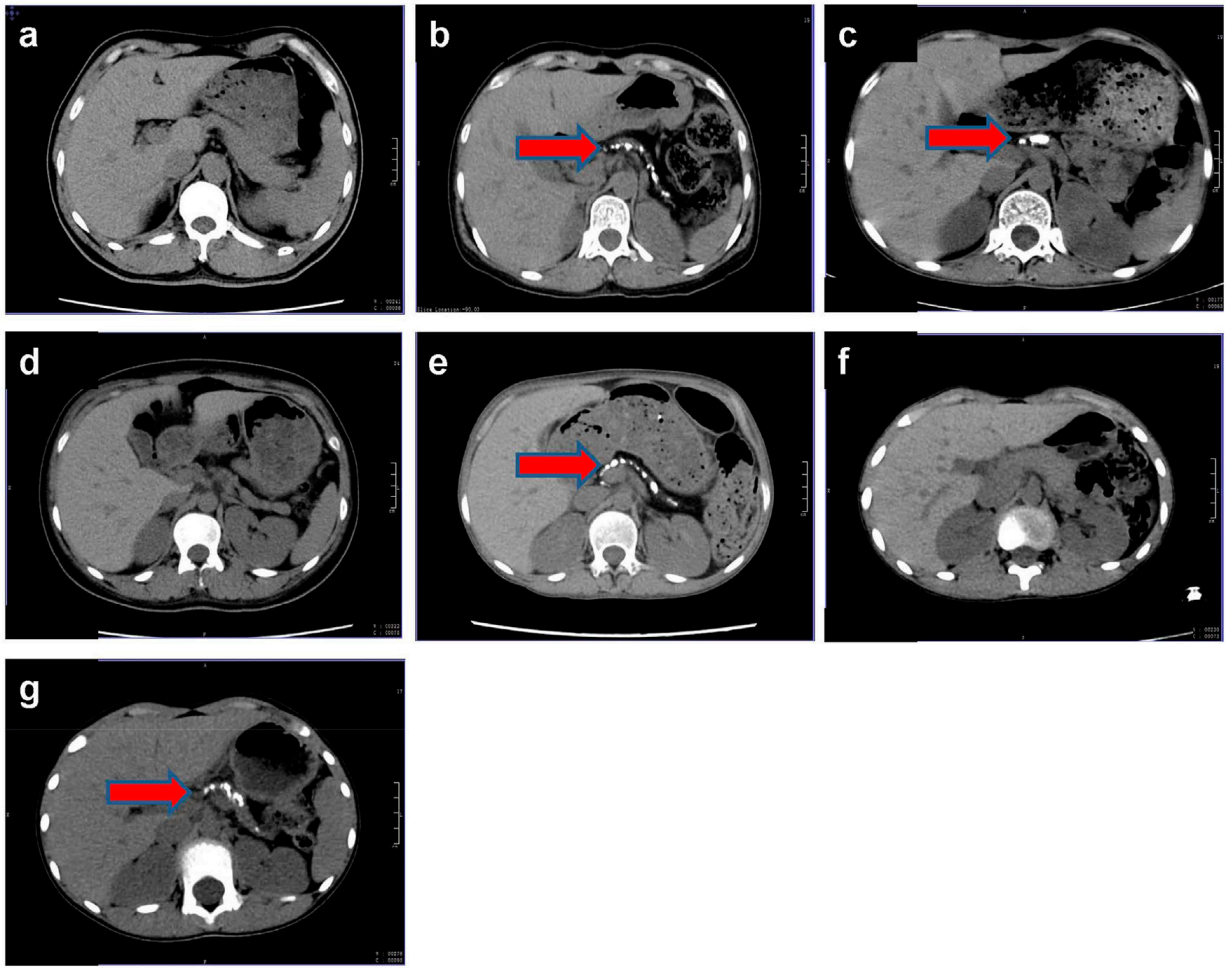
Pancreas CT of this family. **(a)** The proband’s father, Ia; **(b)** the proband’s mother, Ib; **(c)** the proband, IIa; **(d)** the proband’s first sister, IIb; **(e)** the proband’s second sister, IIc; **(f)** the proband’s brother, IId; **(g)** the proband’s third sister, IIe; red arrow, pancreatic calcification.

Laboratory findings for the proband and her family are detailed in Table 1. Additional tests revealed positive urinary glucose but negative urinary ketones. The urinary albumin-to-creatinine ratio was elevated at 112.01 mg/g (normal <30 mg/g). Nerve conduction studies indicated peripheral neuropathy in the right upper limb and both lower limbs. Visual acuity was 0.6 in the right eye and 0.04 in the left eye, with bilateral cataracts observed. CT imaging of the upper abdomen revealed irregular, bead-like dilatation of the pancreatic duct along with marked atrophy and calcification (Figure 2c). Similar imaging findings, although varying in severity, were observed in the proband’s mother and her second and third sisters (Figures 2b,e,g).

**FIGURE 3:**
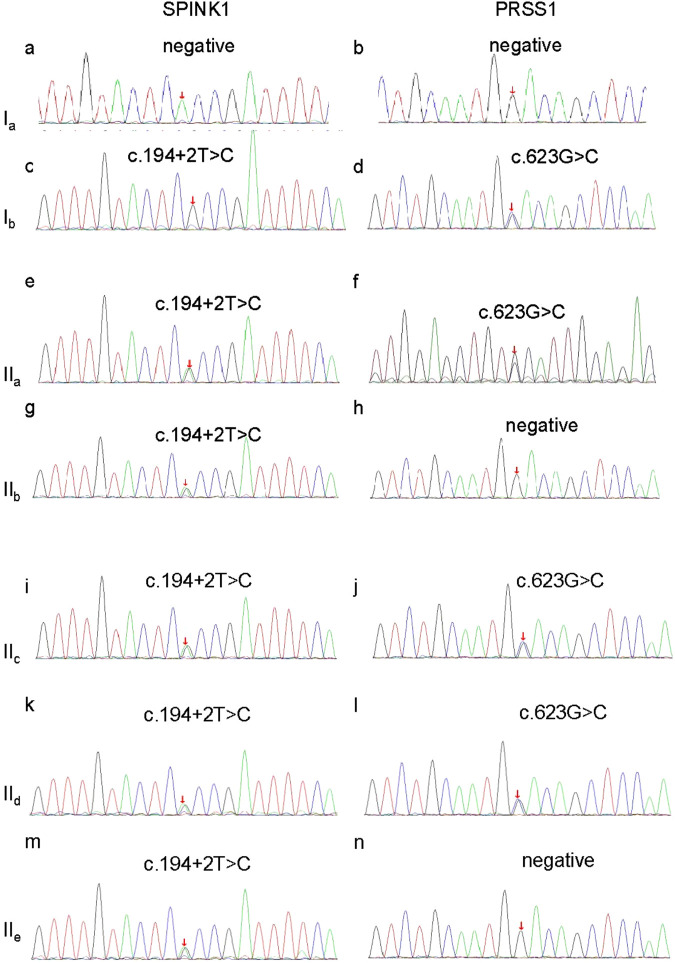
Sanger sequencing verification of the SPINK1 gene and PRSS1 gene in this family. **(a)** No mutation of SPINK1 in Ia; **(b)** no mutation of PRSS1 in Ia; **(c)** c.194+2T>C homozygous mutation of SPINK1 in Ib; **(d)** c.623G>C, heterozygous mutation of PRSS1 in Ib; **(e)** c.194+2T>C heterozygous mutation of SPINK1 in IIa; **(f)** c.623G>C, heterozygous mutation of PRSS1 in IIa; **(g)** c.194+2T>C heterozygous mutation of SPINK1 in IIb; **(h)** no mutation of PRSS1 in IIb; **(i)** c.623G>C, heterozygous mutation of PRSS1 in IIc; **(j)** c.623G>C, heterozygous mutation of PRSS1 in IIc; **(k)** 194+2T>C heterozygous mutation of SPINK1 in IId; **(l)** no mutation of PRSS1 in IId; **(m)** c.194+2T>C heterozygous mutation of SPINK1 in IIe; **(n)** c.623G>C, heterozygous mutation of PRSS1 in IIe; Ia, the proband’s father; Ib, the proband’s mother; IIa, the proband; IIb, the proband’s first sister; IIc, the proband’s second sister; IId, the proband’s brother; IIe, the proband’s third sister.

After obtaining informed consent from the proband and her family, peripheral blood samples were collected from all family members and submitted to MyGenostics Laboratory (Beijing, China) for whole-exome sequencing (WES). This allowed a comprehensive analysis of coding variants across genes known to be associated with pancreatitis. Variant filtering followed standard protocols: variants with a minor allele frequency (MAF) > 1% were excluded, and the remaining variants were annotated using SIFT and PolyPhen-2 and cross-referenced with the Human Gene Mutation Database (HGMD) and ClinVar. Two pathogenic variants were identified and confirmed by Sanger sequencing: a splice-site variant in SPINK1 (c.194+2T>C, ClinVar 132142) and a missense variant in *PRSS1* (c.623G>C, p.G208A, ClinVar 258802). The *SPINK1* variant is heterozygous and results in aberrant splicing, while the *PRSS1* variant is a heterozygous substitution of glycine to alanine. Additional analysis of pancreatitis-associated genes, including CFTR, CASR, CTRC, and TRPV6, revealed no pathogenic or likely pathogenic variants in the proband or her affected relatives. Thus, only the compound heterozygous variants in SPINK1 and PRSS1 were considered clinically significant. The proband (IIa) carried both variants. Her mother (Ib) had a homozygous SPINK1 mutation and a heterozygous PRSS1 mutation. Her second sister (IIc) and brother (IId) carried the same combination as the proband, while her first (IIb) and third (IIe) sisters carried only the SPINK1 variant (Figure 3; Table 1). Both variants were predicted to be damaging by SIFT and PolyPhen-2 and were classified as pathogenic according to the American College of Medical Genetics (ACMG) guidelines. Based on the clinical presentation, laboratory findings, and genetic results, the proband was diagnosed with pancreatogenic diabetes secondary to hereditary pancreatitis.

The proband and her family were followed-up by telephone over 5 years. The proband received comprehensive management, including lifestyle modification, restriction of dietary fat and protein, exogenous pancreatic enzyme replacement, micronutrient and vitamin supplementation, and insulin therapy (aspart before meals and glargine at bedtime). Both the proband and her second sister (IIc) required multiple hospitalizations due to severe glycemic fluctuations and diabetes-related complications. Unfortunately, both died during the follow-up period, although the exact cause of death could not be determined. The proband’s mother (Ib) remained free of abdominal pain or diarrhea, and her mildly elevated blood glucose was controlled with insulin (human insulin before meals and glargine at bedtime). Her first sister (IIb), also treated with aspart and glargine, maintained good glycemic control. Her brother (IId) and third sister (IIe) had not developed diabetes by the end of the follow-up period. However, insulin release testing in IId revealed insulin resistance, indicating a need for continued monitoring to assess potential progression to diabetes (Table 1).

## 3 Discussion

HP is a distinct and uncommon subtype of pancreatitis and is a rare autosomal genetic disease. Its global prevalence remains unclear, although population-based studies from France (Rebours et al., 2009), Denmark (Joergensen et al., 2010), and Australia (Wu et al., 2022) have shown rates of 0.3/100,000, 0.57/100,000, and 1.1/100,000, respectively. Clinically, HP typically manifests in childhood, with recurrent episodes of pancreatitis, abdominal pain, diarrhea, and both exocrine and endocrine pancreatic insufficiency, independent of common risk factors such as alcohol use, gallstones, or trauma (Rebours et al., 2012; Mayerle et al., 2019). Mild diabetes develops in 10% to 25% of affected individuals. Diagnostic criteria include (1) recurrent pancreatitis from early childhood; (2) at least two affected relatives; (3) the presence of pancreatic ductal stones; and (4) exclusion of other known causes such as alcohol, gallstones, trauma, drugs, infections, or metabolic disorders (Raphael and Willingham, 2016).

The pathogenesis of HP involves genetic mutations affecting the regulation of pancreatic digestive enzymes. In 1996, Whitcomb et al. linked HP to mutations in *PRSS1*, which encodes cationic trypsinogen on chromosome 7. Subsequent research identified additional mutations in PRSS2, SPINK1, CTRC, CFTR, and CASR (Suzuki et al., 2020). These mutations promote the premature activation of trypsinogen within the pancreatic ducts, leading to inflammation and autodigestion. Among these, mutations in PRSS1 and SPINK1 are the most frequently implicated in HP. PRSS1 mutations are associated with accelerated progression from acute to chronic pancreatitis and are present in 60%–70% of HP families (Panchoo et al., 2022). In Chinese cohorts, common PRSS1 variants include c.86A>T, c.346C>T, c.364C>T, c.365G>A, and c.623G>C, while SPINK1 variants include c.101A>G and c.194+2T>C(11).

Co-occurrence of multiple gene mutations is relatively rare. In a large Chinese chronic pancreatitis (CP) cohort, 4.34% of idiopathic CP cases harbored both SPINK1 (c.194+2T>C) and PRSS1 (c.623G>C) mutations (Zou et al., 2018). When these two variants co-occur in a heterozygous state, the odds ratio for CP increases significantly (Zou et al., 2018). A previous report also described a patient carrying SPINK1 (c.194+2T>C), PRSS1 (c.623G>C), and STIM1 (p.E152K) (Schnúr et al., 2014), further supporting a synergistic effect among different pathogenic variants. In our case, the proband harbored both SPINK1 (c.194+2T>C) and PRSS1 (c.623G>C) mutations. Multiple lines of evidence support the pathogenicity of these variants. First, both are predicted to be deleterious by SIFT and PolyPhen-2 and are classified as pathogenic per ACMG guidelines. Second, according to gnomAD (accessed 12 May 2025), the allele frequencies are low: 0.0001137 (183/1,609,104) for *SPINK1* c.194+2T>C and 0.0002193 (354/1,614,144) for *PRSS1* c.623G>C. Third, both variants are listed in the HGMD as associated with chronic pancreatitis. Fourth, functionally, SPINK1 c.194+2T>C leads to a loss of SPINK1 function and increased trypsin activation, predisposing the carrier to pancreatitis (Lowenfels et al., 1997; Chavanas et al., 2000). In mouse models, heterozygous SPINK1 c.194+2T>C elevates IL-33 and induces M2 macrophage polarization, activating pancreatic stellate cells and promoting pancreatic fibrosis and chronic inflammation (Liu et al., 2024). Finally, overexpression of PRSS1 c.623G>C in HEK 293 cells, particularly in the presence of CTRC, impairs trypsinogen secretion and promotes misfolding, likely triggering endoplasmic reticulum (ER) stress, which is a known contributor to pancreatitis pathogenesis (Schnúr et al., 2014).

Unlike most HP patients who experience recurrent episodes of acute pancreatitis that progressively evolve into chronic pancreatitis (CP), our proband exhibited only diarrhea attributable to CP despite having extensive pancreatic calcification. This observation suggests that identical genetic mutations can lead to heterogeneous clinical phenotypes. A previous study indicated that the PRSS1 (c.623G>C) mutation may require additional cofactors—genetic, environmental, or lifestyle-related (e.g., alcohol consumption)—to trigger CP development (Masamune et al., 2014). Therefore, we hypothesize that the absence of acute pancreatitis episodes in the proband and her family members may be linked to the lack of such external risk factors influencing the pathogenicity of PRSS1 mutations. Additionally, phenotypic variation was observed within the family. Both the proband and her sibling (IIc) experienced significant glycemic fluctuations and ultimately died during follow-up. In contrast, another family member (IId), who carried the same compound mutation, has not developed diabetes, pancreatitis, or diarrhea to date. These findings underscore the complex pathogenesis of HP, in which clinical outcomes may depend not only on genetic mutations but also on environmental exposures and lifestyle. The proband’s early separation from a stable family environment, poor living conditions, and irregular diet may have exacerbated disease progression. Furthermore, follow-up insulin release tests revealed insulin resistance in IId, suggesting a predisposition to diabetes. Thus, long-term monitoring of IId and IIe remains essential.

HP patients face a 50- to 70-fold increased risk of developing pancreatic cancer (Shelton et al., 2018). While some studies report that only specific mutations, such as p.N34S, are significantly associated with pancreatic cancer risk and thus do not recommend screening for patients with SPINK1 mutations (Greenhalf et al., 2020; Suzuki and Shimizu, 2019), other research suggests that SPINK1 mutation carriers have a 12-fold increased risk compared to controls (Muller et al., 2019). Current clinical guidelines from the American Gastroenterological Association (AGA) recommend initiating pancreatic cancer screening at the age of 40 years in PRSS1 mutation carriers with HP (Aslanian et al., 2020). Although our proband and IIc both died in local hospitals, their causes of death remain unconfirmed, and there is no definitive evidence linking their deaths to pancreatic cancer. Nonetheless, based on existing data, we continue to monitor other mutation-carrying family members for the potential development of diabetes, pancreatitis, and malignancy.

In terms of treatment, HP patients with marked glycemic variability require not only insulin therapy but also dietary management, including reduced fat and protein intake and supplementation with trace elements and vitamins. Those experiencing severe abdominal pain may benefit from analgesics (Rebours et al., 2009; Wu et al., 2022; Lasher et al., 2019). In selected cases, endoscopic or surgical interventions may be indicated. Recent studies have demonstrated that total pancreatectomy with islet cell autotransplantation in a 2-year-old child not only improved quality of life but also reduced the risk of pancreatogenic diabetes and pancreatic cancer after 5 years of follow-up (Marfil-Garza et al., 2021). Gene therapy also holds promise as a future treatment strategy. For instance, Wang et al. (2024) developed an AAV8-hSPINK1 vector targeting the SPINK1 (c.194+2T>C) mutation, which significantly attenuated pancreatitis severity, reduced fibrosis, apoptosis, and autophagy in the pancreas, and promoted recovery in a mouse model, providing a compelling rationale for clinical translation.

Our study has several limitations. First, copy number variations (CNVs) were not assessed due to the limited resolution of the targeted whole-exome sequencing (WES) approach employed. Further evaluation using multiplex ligation-dependent probe amplification (MLPA) or array-based comparative genomic hybridization (array CGH) is needed to rule out CNVs in genes implicated in CP. Second, we did not perform functional studies on the two identified mutations, limiting our ability to assess potential synergistic effects. Third, the intrafamilial variability in clinical presentation cannot be fully explained by environmental differences alone. Additional mechanistic investigations are necessary to elucidate the relationship between the genotype and phenotype in hereditary pancreatitis.

## 4 Conclusion

HP typically presents at an early age and is associated with a high incidence of pancreatic insufficiency and an elevated risk of pancreatic cancer. Pancreatic CT is a routine screening tool used to evaluate pancreatic morphology and the extent of calcification. Genetic screening is recommended to support diagnosis and reduce the likelihood of missed or incorrect diagnoses. Pathogenic variants are most commonly found in the PRSS1 and SPINK1 genes. While diagnosis often relies on family history and genetic testing, there is not always a direct correlation between gene mutations and clinical symptoms, indicating that environmental and lifestyle factors, such as alcohol consumption and physical trauma, may also contribute to disease pathogenesis. Early screening, accurate diagnosis, and multidisciplinary management are critical for patients with HP. In young individuals under 25 years of age who present with diabetes, a lean physique, chronic diarrhea or steatorrhea, poor islet function, and a family history of diabetes—but without classic signs of chronic pancreatitis—HP should be considered in the differential diagnosis. Comprehensive evaluation and integrated management strategies are essential for affected families to improve clinical outcomes and quality of life.

## Data Availability

The original contributions presented in the study are publicly available. This data can be found here: https://ngdc.cncb.ac.cn/bioproject/browse/PRJCA042554.
